# The Diagnostic Performance of Early Sjögren’s Syndrome Autoantibodies in Juvenile Sjögren’s Syndrome: The University of Florida Pediatric Cohort Study

**DOI:** 10.3389/fimmu.2021.704193

**Published:** 2021-06-25

**Authors:** Akaluck Thatayatikom, Inyoung Jun, Indraneel Bhattacharyya, Kathleen Berg, Yun Jong Lee, Yoosik Kim, Abi Adewumi, Weizhou Zhang, Sthorn Thatayatikom, Ankit Shah, Casey Beal, Renee Modica, Melissa E. Elder, Seunghee Cha

**Affiliations:** ^1^ Department of Pediatrics, Division of Allergy, Immunology, Rheumatology, College of Medicine, University of Florida, Gainesville, FL, United States; ^2^ Center for Orphaned Autoimmune Disorders (COAD), College of Dentistry, University of Florida, Gainesville, FL, United States; ^3^ Department of Epidemiology, College of Public Health and Health Professions & College of Medicine, University of Florida, Gainesville, FL, United States; ^4^ Division of Oral Pathology, Department of Oral and Maxillofacial Diagnostic Sciences, College of Dentistry, University of Florida, Gainesville, FL, United States; ^5^ Division of Oral Medicine, Department of Oral and Maxillofacial Diagnostic Sciences, College of Dentistry, University of Florida, Gainesville, FL, United States; ^6^ Division of Rheumatology, Department of Internal Medicine, Seoul National University Bundang Hospital, Seongnam, South Korea; ^7^ Department of Chemical and Biomolecular Engineering, Korea Advanced Institute of Science and Technology (KAIST), Daejeon, South Korea; ^8^ Department of Pediatric Dentistry, College of Dentistry, University of Florida, Gainesville, FL, United States; ^9^ Department of Pathology, Immunology and Laboratory Medicine, College of Medicine, University of Florida, Gainesville, FL, United States; ^10^ Department of Ophthalmology, College of Medicine, University of Florida, Gainesville, FL, United States

**Keywords:** juvenile Sjögren’s syndrome, autoantibodies, early Sjögren’s syndrome autoantibodies, parotitis, minor salivary gland biopsy, sicca symptoms

## Abstract

**Objectives:**

The aim of this study was to evaluate the clinical validity of early Sjögren’s syndrome (SS) autoantibodies (eSjA), which were originally marketed for early diagnosis of SS, for juvenile SS (JSS) in a recently identified pediatric cohort.

**Methods:**

A total of 105 symptomatic subjects with eSjA results available were evaluated at the Center for Orphaned Autoimmune Disorders at the University of Florida and enrolled for this study. JSS diagnosis was based on the 2016 ACR/EULAR SS criteria. Demographic/clinical/laboratory parameters were compared between JSS (n = 27) and non-JSS (n = 78) for % positivity, sensitivity, and specificity of eSjA (SP1, anti-salivary protein; CA6, anti-carbonic anhydrase VI; PSP, anti-parotid secretory protein) and classic SS-autoantibodies (cSjA; ANA, SSA/SSB, RF, and others) either alone or in combination. Associations between eSjA and diagnostic/glandular parameters were also determined by Fisher’s exact test.

**Results:**

Compared to non-JSS, JSS patients exhibited sicca symptoms demonstrating reduced unstimulated salivary flow rate (USFR) and abnormal glandular features revealed by salivary gland ultrasound (SGUS). Among cSjA, ANA demonstrated the highest sensitivity of 69.2%, while SSA, SSB, and RF showed around 95% specificities for JSS diagnosis. The % positive-SSA was notably higher in JSS than non-JSS (56% vs. 5%). Of eSjA, anti-CA6 IgG was the most prevalent without differentiating JSS (37%) from non-JSS (32%). Sensitivity and specificity of eSjA were 55.6 and 26.9%, respectively. Autoantibodies with potentially applicable specificity/sensitivity for JSS were seen only in cSjA without a single eSjA included. There were no associations detected between eSjA and focus score (FS), USFR, SSA, SGUS, and parotitis/glandular swelling analyzed in the entire cohort, JSS, and non-JSS. However, a negative association between anti-PSP and parotitis/glandular swelling was found in a small group of positive-SSA (n = 19, p = 0.02) whereas no such association was found between anti-PSP-positive compared to anti-PSP-negative. JSS and non-JSS groups differed in FS, USFR, and EULAR SS Patient Reported Index Dryness/Mean in CA6/PSP/ANA, SP1, and SSA-positive groups, respectively. Additionally, a higher FS was found in RF-positive than RF-negative individuals.

**Conclusions:**

eSjA underperformed cSjS in differentiating JSS from non-JSS. The discovery of clinical impact of eSjA on early diagnosis of JSS necessitates a longitudinal study.

## Introduction

Sjögren’s syndrome (SS) is an autoimmune disorder that typically leads to sicca symptoms of severe dry eyes and dry mouth, mainly in female patients with peak incidence between 45 and 55 years of age (1). Sicca symptoms are believed to be less prevalent in juvenile SS (JSS) compared to adult SS patients (2–9). Recurrent parotitis in JSS is one of the main symptoms that warrants referral to rheumatologists for care. As longitudinal follow-up studies on JSS patients are absent, it is unknown if JSS will progress to adult SS. Moreover, it is not clear whether JSS patients will develop the major complication of non-Hodgkin’s B-cell lymphoma reported to occur in 5% of affected adult patients (1) or if JSS is a distinct disease entity with a unique clinical course.

Currently, there are no consensus diagnostic criteria tailored specifically to pediatric patients with JSS. The 2002 American-European Consensus Group (AECG) criteria (10), 2012 American College of Rheumatology (ACR) classification criteria (11), or 2016 ACR/EULAR classification criteria (12) for adult SS have been used in most JSS case studies and reports due to lack of validated criteria in children. According to the most recent 2016 ACR/EULAR criteria, serology for anti-SSA/Ro (SSA), minor salivary gland (MSG) lip biopsy for focus score, sialometry for unstimulated saliva flow rate (USFR), Schirmer’s test, and ocular surface staining (OSS) are included for diagnosis. SSA positivity and positive MSG biopsy are each assigned a weight of three points, while USFR, Schirmer’s test, and OSS are assigned one point per item. When summation is greater than or equal to four points, SS diagnosis is established. However, subjective sicca symptoms are not a part of the current SS diagnostic criteria (12). Since there are different characteristics noted in JSS from SS, such as the prevalent recurrent parotitis mentioned earlier, use of the adult SS classification or diagnostic criteria may lead to misdiagnosis or underrepresentation of JSS.

In recent years, Shen et al. reported a panel of autoantibodies that were detected in their IL-14α transgenic mice (IL14α TG) with sialadenitis and in a group of SS patients (13). Because these autoantibodies were more prevalent in SSA-negative adult SS patients or in relatively newly diagnosed adult sicca patents, Shen et al. proposed them as early biomarkers for SS. These antibodies were later commercialized by Trinity Biotech under the name of “early SS autoantibody (eSjA) panel” and screened by their Immco Diagnostics Reference Lab. The panel includes three autoantibodies, namely salivary protein 1 (SP1), carbonic anhydrase IV (CA6), and parotid secretory protein (PSP), with three immunoglobulin isotypes (IgG, IgA, and IgM) for each antibody, totaling nine items.

In addition, the same company produced the Sjö^®^ test kit, which combines three in eSjA with the four classic SS autoantibodies (cSjA), ANA, SSA, anti-SSB/La (SSB), and rheumatoid factor (RF), encompassing seven SS biomarkers (Immco.com). The sensitivity and specificity of eSjA in differentiating SS from non-SS are unclear as most publications on eSjA report % prevalence rather than sensitivity and specificity. The company website lists the prevalence of eSjA as 30–45% in SS. The sensitivity and specificity of the Sjö^®^ test kit in SS diagnosis reported in two 2016 conference abstracts indicated the cumulative sensitivity of the Sjö test kit was 91.4%, the sensitivity of SSA/SSB was 74.9%, and the sensitivity for eSjA was 49.8%. The cumulative specificity for the Sjö test kit was 79.8% and the specificity for eSjA was 83.5% while the specificity for SSA/SSB was not listed (14, 15). However, the details on demographics, diagnostic criteria, and clinical parameters of the cohort investigated were unavailable.

In this study, we determined if eSjA could differentiate JSS from non-JSS in patients with sicca symptoms based on the assumption that JSS could be an early manifestation of SS. We examined cSjA as well as eSjA in pediatric patients presenting to the Pediatric Rheumatology Clinic at the University of Florida (UF). To our knowledge, this is the first and only study reporting the levels of sensitivity and specificity of eSjA in differentiating JSS from non-JSS patients, who did not meet the 2016 ACR/EULAR SS criteria.

## Patients and Methods

### Subject Enrollment

Participants were recruited from January, 2018 to November, 2020, at the Oral Medicine clinic, College of Dentistry, and the Pediatric Rheumatology clinic, College of Medicine, at the UF Health Shands Hospital, Gainesville, Florida. Enrollment was made during a routine medical check-up. JSS patients were diagnosed according to the 2016 ACR/EULAR criteria, and patients with sicca symptoms who did not fulfil the criteria were classified as non-JSS. Only those who had been tested for eSjA (n = 105) were included in the analysis. Patients in the JSS group (n = 27, F:M = 21:6) ranged from 4 to 27 years of age, including one 20 and one 27 year-old. Ages of non-JSS subjects (n = 78, F:M = 54:24) ranged from 5 to 20, including two 18 year-old and one 20 year-old (**Table 1**). Those subjects older than 18 were included because their symptoms started before 18 years of age and lasted until they were evaluated at the Pediatric Rheumatology Clinic without having been evaluated for JSS elsewhere. Primary and secondary JSS were not classified due to the lack of guidelines for the JSS classification criteria at the present time. This study was reviewed and approved by the Institutional Review Board (IRB protocols #201600490 and 201900645) at UF and written informed consents/assents were obtained from either parents/guardians of pediatric patients or participants who were older than 18 years of age.

**Table 1:** Demographic, clinical, and laboratory characteristics of the UF pediatric study cohort.

	JSS (N = 27)	Non-JSS (N = 78)	p-value
***General***			
Age, symptom onset (Median, IQR)	11 (5.5)	11 (6.75)	0.478
Age, diagnosis (Median, IQR)	14 (6)	14 (6.25)	0.570
Female	21 (77.8%)	54 (69.2%)	0.466
***Laboratory parameters***			
ESR elevated	8/26 (30.8%)	9/75 (12.0%)	0.09
CRP elevated	5/26 (19.2%)	13/74 (17.6%)	0.999
C3 low	2/24 (8.3%)	2/66 (3.0%)	0.408
C4 low	3/24 (12.5%)	7/65 (10.8%)	0.805
Hypergammaglobulinemia	10/27 (37.0%)	5/78 (6.4%)	<0.001**
Various autoantibodies	Tables 2 & 3	Tables 2 & 3	–
Cytopenia (neutropenia, lymphopenia, thrombocytopenia)	8/27 (29.6%)	8/78 (10.3%)	0.027*
Autoimmune hemolytic anemia	0/27 (0%)	4/78 (5.1%)	0.57
***Diagnostic tests***			
Schirmer’s test	5/16 (31.3%)	6/42 (14.3%)	0.275
Unstimulated salivary flow rate (USFR)	11/27 (40.7%)	10/78 (12.8%)	0.004**
Minor salivary gland lip biopsy (MSGBx)	22/26 (84.6%)	18/47 (38.3%)	<0.001**
Salivary gland ultrasound (SGUS)	16/26 (61.5%)	9/58 (15.5%)	<0.001**
***Medical history***			
Infection (Bacterial, viral, and/or fungal)	11 (40.7%)	25 (32.1%)	0.483
Various immune-related conditions^&^	13 (48.1%)	40 (51.3%)	0.826
ADHD or Autism	2 (7.4%)	10 (12.8%)	0.727
Recurrent/persistent parotitis or glandular swelling/tenderness	19 (70.4%)	33 (42.3%)	0.015*
***Clinical features***			
Dry eyes (subjective)	21 (77.8%)	45 (57.7%)	0.069
Dry mouth (subjective)	24 (88.9%)	55 (70.5%)	0.071
Dry eyes and dry mouth (subjective)	20 (74.1%)	38 (48.7%)	0.026*
Dysphagia	4 (14.8%)	17 (21.8%)	0.58
Dry lips	11 (40.7%)	43 (55.1%)	0.264
Oral ulcers	13 (48.1%)	24 (30.8%)	0.16
TMD or TMJ clicking	8 (29.6%)	16 (20.5%)	0.425
Dental issues	16 (59.3%)	23 (41.8%)	0.01*
Hypermobile joints	17 (63.0%)	50 (64.1%)	0.999
***ESSDAI/ESSPRI-related domains and other features***			
ESSPRI Dryness (Median, IQR)	5	4 (6)	0.565
ESSPRI Fatigue (Median, IQR)	5.5 (7.25)	5 (5)	0.824
ESSPRI Pain (Median, IQR)	1 (6)	3 (5)	0.797
ESSPRI mean (Median, IQR)	4.17 (3.41)	3.33 (4)	0.880
Fever	8 (29.6%)	16 (20.5%)	0.425
Weight loss	4 (14.8%)	5 (6.4%)	0.231
Lymphadenopathy or lymphoma	4 (14.8%)	13 (16.7%)	0.999
Cutaneous	1 (3.7%)	1 (1.3%)	0.45
Pulmonary	14 (51.9%)	46 (59.0%)	0.652
Renal	7 (25.9%)	4 (5.1%)	0.006**
Muscular	14 (51.9%)	44 (56.4%)	0.823
Articular	23 (85.2%)	66 (84.6%)	0.999
Neurological	22 (81.5%)	65 (83.3%)	0.999
Cardiovascular	5 (18.5%)	16 (20.5%)	0.999
Gastrointestinal	13 (48.1%)	44 (56.4%)	0.506
Skin	17 (63.0%)	54 (69.2%)	0.635
Raynaud’s	4 (14.8%)	7 (9.0%)	0.468

Bold, ***p < 0.001, **p < 0.01 and *p < 0.05; ESSDAI, EULAR SS Disease Activity Index; ESSPRI, EULAR SS Patient Reported Index. ^&^SLE (systemic lupus erythematosus), juvenile idiopathic arthritis, Crohn’s disease, Immune deficiency, amplified pain syndrome, type I diabetes, fibromyalgia, Hashimoto’s disease, anti-phospholipid syndrome, IgG4 disease, mixed connective tissue disease, or systemic sclerosis.

### Clinical and Laboratory Parameters

Laboratory results were collected from the Center for Orphaned Autoimmune Disorders (COAD) tissue bank and patient registry, following the current diagnostic guidelines for adult SS (12, 16). USFR was measured by allowing saliva to flow naturally into a preweighed vessel for 10 min. Flow rates below or equal to the cut-off of 0.1ml/min are considered indicative of hyposalivation. Dry eye condition was mainly evaluated by a combination of slit lamp examination, conjunctival and corneal staining patterns, tear break-up time, and Schirmer’s test. The Schirmer’s test, utilizes sterile strips (TearFlo, Sigma Pharmaceuticals, Monticello, CA) placed in the inferior fornix of the patient to measure basal tear production for 5 min with topical anesthesia as children do not usually tolerate the strips without it. A value less than 5 mm of tear production at 5 min constituted severe dry eyes. OSS was evaluated in only a small number of patients and therefore was not included in the analysis. MSG biopsy results were reported positive if there was one or more foci of at least 50 immune cells per 4 mm^2^ of salivary gland tissue. Salivary gland ultrasound (SGUS) of bilateral parotid and submandibular glands was classified as positive when the simplified score was 2 or 3, which is suggestive of SS (17). Test results for cSjA, eSjA, WBC, ESR, CRP, C3 and C4, total IgG, and CBC were collected within 2 weeks of enrollment. ANA was coded positive at titers of 1:160 or greater, and eSjA positivity was determined based on the laboratory report by Immco Diagnostics (Buffalo, NY), which includes nine items; IgG, IgA, and IgM of anti-SP1, anti-CA6, and anti-PSP. Items in the twelve domains of the physician-reported EULAR SS disease activity index (ESSDAI) and the EULAR SS patient reported index (ESSPRI) including dryness, somatic and mental fatigue, and pain indices (16, 18) were also analyzed in this study.

### Statistical Analyses

Group comparisons between JSS and non-JSS were performed on dichotomous variables (e.g., positive/negative) with the Fisher’s exact test. For continuous variables, such as age at symptom onset, age at diagnosis, ESSPRI Dryness, ESSPRI Fatigue, ESSPRI Pain, and ESSPRI Mean, the non-parametric Mann–Whitney–Wilcoxon test was performed. A p-value of less than 0.05 was considered significant for both analyses. The % positivity along with sensitivity and specificity were calculated at both the level of single autoantibodies and autoantibodies in combination in order to explore the diagnostic performance. A performance graph was prepared for easy comparison between eSjA and cSjA in distinguishing JSS from non-JSS in patients with xerostomia. Associations with odds ratio and confidence intervals (CI) between eSjA and the items in the 2016 ACR/EULAR SS diagnostic criteria, such as FS, SSA, USFR, and the Schirmer’s tear test, and the items reflecting glandular features, such as recurrent parotitis/glandular swelling and SGUS, were calculated for the category of the entire study cohort, JSS, non-JSS, positive-SSA, or negative-SSA group. In each category, we evaluated the association between diagnostic/glandular items with eSjA, using Fisher’s exact test. We further compared JSS with non-JSS, or antibody-positive group with antibody-negative group using the Mann–Whitney–Wilcoxon test. Mean diagnostic values were presented in the bar plots with dots representing the distribution of those values. All analyses were calculated by R (http://www.r-project.org, version 4.0.3) in RStudio (http://www.rstudio.com, version 1.4.1106).

## Results

### JSS Patients Exhibit History of Sicca Symptoms and Glandular Changes Noted by SGUS

**Table 1** summarizes demographic, clinical, and laboratory characteristics of the eSjA study population (n = 105) including both JSS (n = 27) and non-JSS patients (n = 78). The median age for symptom onset was around 11 years old in both groups, with a 2 year old boy being the youngest JSS patient in our cohort. In general, it took about 3 years from symptom onset to be either diagnosed or ruled out as JSS in our cohort. Of 27 JSS patients, 21 were female (77.8%).

Of laboratory parameters measured, hypergammaglobulinemia and cytopenias differed significantly between JSS and non-JSS groups (p < 0.001 and p = 0.027, respectively). Differences between JSS and non-JSS groups for diagnostic evaluations of USFR (p = 0.004), MSG biopsy (p < 0.001), and SGUS (p < 0.001) were also significant, along with patient history of experiencing recurrent/persistent parotitis and/or glandular swelling/tenderness (p = 0.015). The number of patients who had both dry eyes and dry mouth (sicca symptoms) was statistically higher in JSS than in non-JSS (p = 0.026). Similarly, dental caries or dental-related issues were also more prevalent in JSS patients (p = 0.01). Interestingly, about 11% of our cohort patients reported attention-deficit/hyperactive disorder (ADHD) or autism spectrum disorders, suggesting a potential link between maternal autoimmune diseases/inflammation and those conditions (19, 20).

In general, ESSDAI domains or ESSPRI items listed in **Table 1** did not show much difference between the two groups except for the biological domain mentioned above and the renal domain (p = 0.006). The test results of some parameters, such as the renal domain or Raynaud’s phenomenon, were available only in a small number of subjects.

### Among cSjA, ANA Shows the Highest Sensitivity While Others Are High in Specificity for JSS

Next, we evaluated the performance of each cSjA alone and in combination to determine which were most effective in distinguishing JSS from non-JSS (**Table 2**). Among 105 study subjects, most patients had blood results for ANA (n = 98, 93%), SSA (n = 103, 98%), and SSB (n = 102, 97%), while only 64% of the study population had been tested for RF. The % positive ANA in the study cohort was 40% with the sensitivity and specificity being 69.2 and 70.8%, respectively.

**Table 2:** Diagnostic performance of cSjA for JSS diagnosis.

	Tested, N (%)	JSS pos, n (%)	Non-JSS pos, n (%)	Total pos, n (%)	Sensitivity	Specificity
***Single***						
ANA	98 (93%)	18 (69%)	21 (29%)	39 (40%)	69.2	70.8
SSA (Ro)	103 (98%)	15 (56%)	4 (5%)	19 (18%)	55.6	94.7
SSB (La)	102 (97%)	4 (15%)	3 (4%)	7 (7%)	14.8	96
RF	67 (64%)	7 (33%)	2 (4%)	9 (13%)	33.3	95.7
CCP	35 (33%)	1 (8%)	0 (0%)	1 (3%)	7.7	ND
dsDNA	79 (75%)	4 (17%)	5 (9%)	9 (11%)	16.7	90.9
Sm	82 (78%)	2 (8%)	3 (5%)	5 (6%)	7.7	94.6
RNP	71 (68%)	5 (21%)	4 (9%)	9 (13%)	20.8	91.5
TPO	68 (65%)	2 (11%)	7 (14%)	9 (13%)	10.5	85.7
Thyroglobulin	63 (60%)	2 (11%)	4 (9%)	6 (10%)	11.1	91.1
Mitochondrial	68 (65%)	1 (5%)	3 (7%)	4 (6%)	4.6	93.5
Centromere	70 (67%)	2 (9%)	1 (2%)	3 (4%)	8.7	97.9
Cardiolipin IgG	36 (34%)	1 (9%)	1 (4%)	2 (6%)	9.1	96
Cardiolipin IgM	33 (31%)	0 (0%)	2 (9%)	2 (6%)	ND	91.3
Beta-2 glycoprotein1 IgG	37 (35%)	1 (8%)	2 (8%)	3 (8%)	8.3	92
Beta-2 glycoprotein1 IgM	37 (35%)	0 (0%)	1 (4%)	1 (3%)	ND	96
Phospholipid IgG	5 (5%)	ND	2 (40%)	2 (40%)	ND	60
Phospholipid IgM	8 (8%)	ND	1 (12%)	1 (12%)	ND	87.5
Histone	9 (9%)	1 (25%)	2 (40%)	3 (33%)	25	60
***Combination (at least one positive)***						
ANA/SSA (ANA **or** SSA)	105 (100%)	21 (78%)	22 (28%)	43 (41%)	77.8	71.8
ANA/SSB	105 (100%)	18 (67%)	22 (28%)	40 (38%)	66.7	71.8
ANA/RF	101 (96%)	18 (69%)	23 (31%)	41 (41%)	69.2	69.3
SSA/SSB	104 (99%)	15 (56%)	6 (8%)	21 (20%)	55.6	92.2
SSA/RF	103 (98%)	16 (59%)	6 (8%)	22 (21%)	59.3	92.1
SSB/RF	103 (98%)	7 (26%)	5 (7%)	12 (12%)	25.9	93.4
ANA/SSA/SSB	105 (100%)	21 (78%)	23 (29%)	44 (42%)	77.8	70.5
ANA/SSA/RF	105 (100%)	21 (78%)	24 (31%)	45 (43%)	77.8	69.2
SSA/SSB/RF	104 (99%)	16 (59%)	8 (10%)	24 (23%)	59.3	89.6
ANA/SSA/SSB/RF	105 (100%)	21 (78%)	25 (32%)	46 (44%)	77.8	68.0
***Combination (all positive)***						
ANA + SSA (ANA **and** SSA)	96 (91%)	12 (46%)	3 (4%)	15 (16%)	46.2	95.7
ANA + SSB	95 (90%)	4 (15%)	2 (3%)	6 (6%)	15.4	97.1
ANA + RF	64 (61%)	7 (33%)	0 (0%)	7 (11%)	33.3	ND
SSA + SSB	101 (96%)	4 (15%)	1 (1%)	5 (5%)	14.8	98.7
SSA + RF	67 (64%)	6 (29%)	0 (0%)	6 (9%)	28.6	ND
SSB + RF	66 (63%)	4 (19%)	0 (0%)	4 (6%)	19.1	ND
ANA + SSA + SSB	94 (90%)	4 (15%)	1 (1%)	5 (5%)	15.4	98.5
ANA + SSA + RF	64 (61%)	6 (29%)	0 (0%)	6 (9%)	28.6	NA
SSA + SSB + RF	66 (63%)	4 (19%)	0 (0%)	4 (6%)	19.1	ND
ANA + SSA + SSB + RF	63 (60%)	4 (19%)	0 (0%)	4 (6%)	19.1	ND

P-values between JSS and non-JSS were not calculated due to a small sample number size. The specificity and sensitivity of each autoantibody were calculated based on the total number of positive cSjA. ND, not-determined because antibody positive subject was absent in either group.

SSA showed relatively high sensitivity and specificity of 55.6 and 94.7%, respectively, for JSS. The % positive SSA was noticeably higher in the JSS group than the non-JSS group (56% *vs*. 5%), as was the % positivity of RF (33% *vs*. 4%). Many autoantibodies, such as SSB, anti-dsDNA, anti-RNP, anti-thyroid peroxidase (TPO), and others including anti-mitochondrial, anti-centromere, and anti-cardiolipin IgG, which are generally seldom reported in pediatric populations, showed high specificity, but the sensitivities of those were significantly low (**Table 2**).

When we considered either ANA positivity or SSA positivity (ANA/SSA), we observed 77.8% sensitivity and 71.8% specificity for JSS distinguished from non-JSS. These values for ANA/RF or ANA/SSA/RF were similar. If a patient had both positive ANA and positive SSA (ANA + SSA), the sensitivity decreased to 46.2%, but the specificity increased to 95.7%. All other combinations showed relatively low sensitivity. Overall, the percentages of a single positive ANA, SSA, SSB, or RF were higher in the JSS group compared to the non-JSS group. Some values could not be calculated (ND in **Table 2**) because all patients in either group were negative for a given autoantibody.

### Anti-CA6 IgG Is Most Prevalent Among eSjA Without Distinguishing JSS From Non-JSS

The % positivity of eSjA is summarized in **Table 3**. Approximately 52% of the total subjects were positive for at least one of the anti-CA6 antibodies. In JSS, anti-CA6 IgG was most prevalent (37%), followed by anti-CA6 IgM (22%), anti-SP1 IgM (19%), and anti-PSP IgG (15%)/IgA (15%). The rank of % positivity was similar in non-JSS, showing anti-CA6 IgG (32%), anti-CA6 IgM (23%), anti-PSP IgG (18%), and anti-SP1 IgG (14%). Overall, anti-CA6 IgG/IgM were most prevalent in our study population without showing a difference in % positivity between JSS and non-JSS.

**Table 3:** Diagnostic performance of eSjA for JSS diagnosis.

	Tested n (%)	JSS pos, n (%)	Non-JSS pos, n (%)	Total pos, n (%)	Sensitivity	Specificity
***Single***						
SP1 (at least one positive)	105 (100%)	7 (26%)	25 (32%)	32 (30%)	25.9	68.0
SP1 IgG	105 (100%)	1 (4%)	11 (14%)	12 (11%)	3.7	85.9
SP1 IgA	105 (100%)	2 (7%)	8 (10%)	10 (10%)	7.4	89.7
SP1 IgM	105 (100%)	5 (19%)	10 (13%)	15 (14%)	18.5	87.2
CA6 (at least one positive)	105 (100%)	13 (48%)	42 (54%)	55 (52%)	48.2	46.2
CA6 IgG	105 (100%)	10 (37%)	25 (32%)	35 (33%)	37.0	68.0
CA6 IgA	105 (100%)	2 (7%)	2 (3%)	4 (4%)	7.4	97.4
CA6 IgM	105 (100%)	6 (22%)	18 (23%)	24 (23%)	22.2	76.9
PSP (at least one positive)	105 (100%)	7 (26%)	26 (33%)	33 (31%)	25.9	66.7
PSP IgG	105 (100%)	4 (15%)	14 (18%)	18 (17%)	14.8	82.1
PSP IgA	105 (100%)	4 (15%)	9 (12%)	13 (12%)	14.8	88.5
PSP IgM	105 (100%)	1 (4%)	6 (8%)	7 (7%)	3.7	92.3
***^#^Combination (at least one positive)***						
SP1/CA6 (SP1 **or** CA6)	105 (100%)	14 (52%)	53 (68%)	67 (64%)	51.9	32.1
SP1/PSP	105 (100%)	13 (48%)	37 (47%)	50 (48%)	48.2	52.6
CA6/PSP	105 (100%)	14 (52%)	49 (63%)	63 (60%)	51.9	37.2
SP1/CA6/PSP	105 (100%)	15 (56%)	57 (73%)	72 (69%)	55.6	26.9
ANA/eSjA	105 (100%)	22 (81%)	63 (81%)	85 (81%)	81.5	19.2
SSA/eSjA	105 (100%)	22 (81%)	58 (74%)	80 (76%)	81.5	25.6
SSB/eSjA	105 (100%)	16 (59%)	58 (74%)	74 (70%)	59.3	25.6
RF/eSjA	105 (100%)	18 (67%)	58 (74%)	76 (72%)	66.7	25.6
ANA/SSA/eSjA	105 (100%)	24 (89%)	63 (81%)	87 (83%)	88.9	19.2
ANA/SSB/eSjA	105 (100%)	22 (81%)	63 (81%)	85 (81%)	81.5	19.2
ANA/RF/eSjA	105 (100%)	22 (81%)	64 (82%)	86 (82%)	81.5	18.0
SSA/SSB/eSjA	105 (100%)	22 (81%)	59 (76%)	81 (77%)	81.5	24.4
SSA/RF/eSjA	105 (100%)	23 (85%)	59 (76%)	82 (78%)	85.2	24.4
SSB/RF/eSjA	105 (100%)	18 (67%)	59 (76%)	77 (73%)	66.7	24.4
ANA/SSA/SSB/eSjA	105 (100%)	24 (89%)	63 (81%)	87 (83%)	88.9	19.2
ANA/SSA/RF/eSjA	105 (100%)	24 (89%)	64 (82%)	88 (84%)	88.9	18.0
ANA/SSB/RF/eSjA	105 (100%)	22 (81%)	64 (82%)	86 (82%)	81.5	18.0
SSA/SSB/RF/eSjA	105 (100%)	22 (81%)	60 (77%)	82 (78%)	81.5	23.1
ANA/SSA/SSB/RF/eSjA	105 (100%)	24 (89%)	64 (82%)	88 (84%)	88.9	18.0
***^#^Combination (all positive)***						
SP1 + CA6 (SP1 **and** CA6)	105 (100%)	6 (22%)	14 (18%)	20 (19%)	22.2	82.1
SP1 + PSP	105 (100%)	1 (4%)	14 (18%)	15 (14%)	3.7	82.1
CA6 + PSP	105 (100%)	6 (22%)	19 (24%)	25 (24%)	22.2	75.6
SP1 + CA6 + PSP	105 (100%)	1 (4%)	11 (14%)	12 (11%)	3.7	85.9
ANA + eSjA	98 (93%)	11 (42%)	15 (21%)	26 (27%)	42.3	79.2
SSA + eSjA	103 (98%)	8 (30%)	3 (4%)	11 (11%)	29.6	96.1
SSB + eSjA	102 (97%)	3 (11%)	2 (3%)	5 (5%)	11.1	97.3
RF + eSjA	67 (64%)	4 (19%)	1 (2%)	5 (7%)	19.1	97.8
ANA + SSA + eSjA	96 (91%)	7 (27%)	2 (3%)	9 (9%)	26.9	97.1
ANA + SSB + eSjA	95 (90%)	3 (12%)	1 (1%)	4 (4%)	11.5	98.6
ANA + RF + eSjA	64 (61%)	4 (19%)	0 (0%)	4 (6%)	19.1	ND
SSA + SSB + eSjA	101 (96%)	3 (11%)	1 (1%)	4 (4%)	11.1	98.7
SSA + RF + eSjA	67 (64%)	4 (19%)	0 (0%)	4 (6%)	19.1	ND
SSB + RF + eSjA	66 (63%)	3 (14%)	0 (0%)	3 (5%)	14.3	ND
ANA + SSA + SSB + eSjA	94 (90%)	3 (12%)	1 (1%)	4 (4%)	11.5	98.5
NA + SSA + RF + eSjA	64 (61%)	4 (19%)	0 (0%)	4 (6%)	19.1	ND
ANA + SSB + RF + eSjA	63 (60%)	3 (14%)	0 (0%)	3 (5%)	14.3	ND
ANA + SSA + SSB + RF + eSjA	63 (60%)	3 (14%)	0 (0%)	3 (5%)	14.3	ND

P-values were not calculated due to a small sample number size in each group. The specificity and sensitivity of each autoantibody were calculated based on the total number of two groups. ^#^eSjA was counted positive when at least one of nine autoantibodies was positive. ND, not determined due to absence of antibody-positive subject in either group.

For all single items of eSjA, the sensitivity was low (3.7–37.0%) while specificity was relatively high (68.0–97.4%). When an analysis was made on combinations of the antibodies to be more inclusive, the sensitivity increased while the specificity decreased significantly. For instance, 81% of our study population had either positive ANA or eSjA (ANA/eSjA, at least one of the nine items being positive for the latter). This combination resulted in 81.5% sensitivity and 19.2% specificity. Similarly, SSA or eSjA (SSA/eSjA) showed 81.5% sensitivity and 25.6% specificity. When at least one of the nine items in eSjA was positive (SP1/CA6/PSP), the sensitivity and specificity became 55.6 and 26.9%, respectively.

When we analyzed the autoantibodies in combination, the sensitivity decreased while the specificity increased as expected. For example, the sensitivity decreased to 3.7% while the specificity increased to 85.9% when all three groups of eSjA were positive (SP1 + CA6 + PSP) with at least one positive item in each group. Twenty-seven percent of our study population had both positive ANA and eSjA (ANA + eSjA), which, in combination, yielded a sensitivity of 42.3% and specificity of 79.2%. With SSA, the change was even more pronounced, with a sensitivity of 29.6% while specificity increased to 96.1%.

### One Cluster Containing cSjA Shows Both Sensitivity and Specificity Above 65% Whereas eSjA Presents High Variability in Specificity or Sensitivity

**Figure 1:**
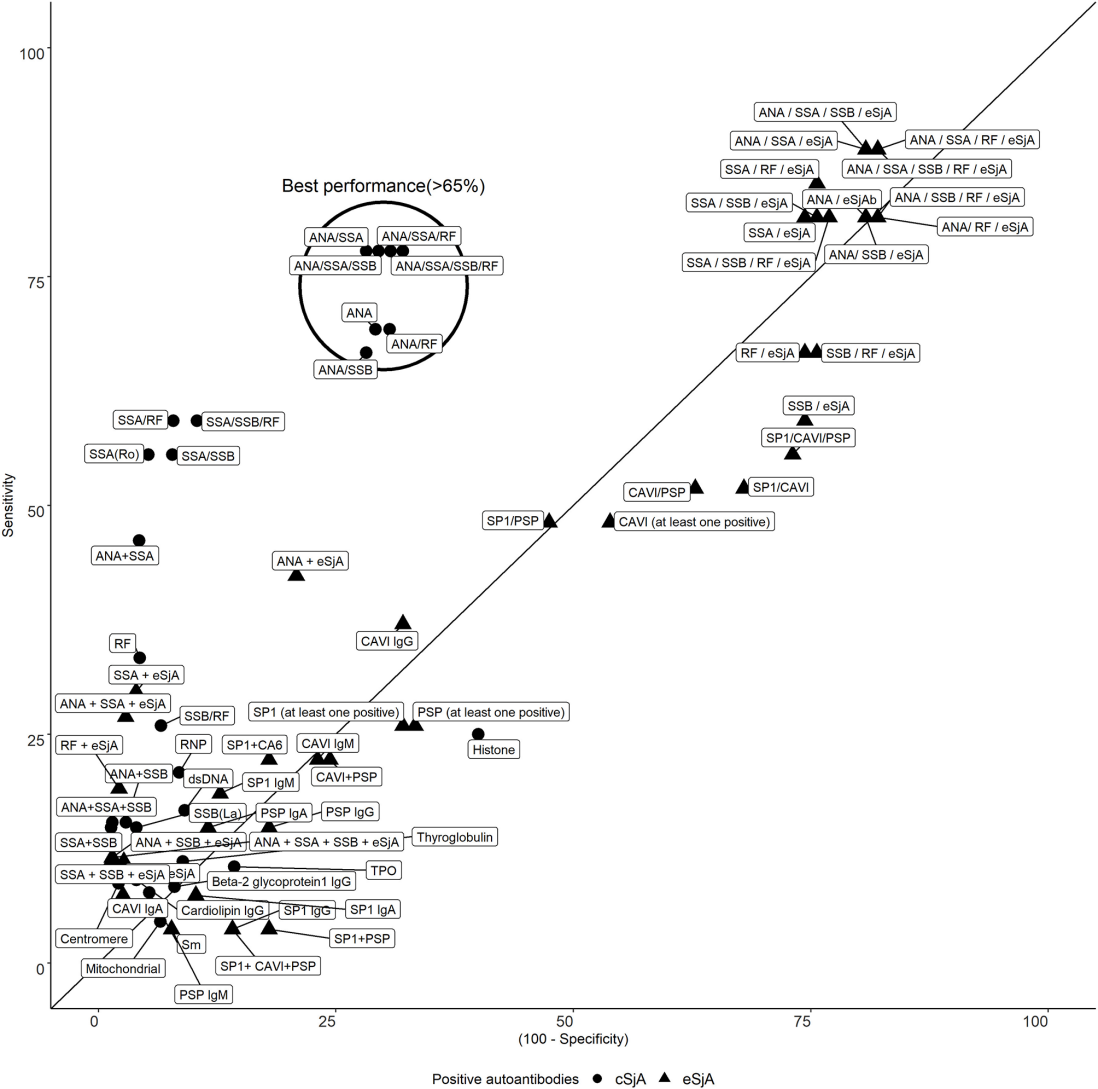
Sensitivity and specificity of cSjA or eSjA in single/combination. The points within or near the circle indicate the best performance as having both sensitivity and specificity above 65%. No eSjA points are found in this cluster.

There was a clear trade-off between sensitivity and specificity of single/combination of autoantibodies as mentioned earlier. In **Figure 1**, most of the eSjA points are located either in the areas of high sensitivity and low specificity, or the areas of low sensitivity with high specificity. Points corresponding to autoantibodies in cSjA, such as ANA, ANA/SSA, or ANA/RF, fall within the circled cluster, with both sensitivity and specificity above 65%. This cluster shows the best performance in distinguishing JSS from non-JSS in **Figure 1**. No single eSjA was included in this cluster.

### Anti-PSP Is Negatively Associated With Parotitis/Glandular Swelling in SSA-Positive JSS

To determine the association between eSjA and diagnostic/glandular parameters, such as MSG lip biopsy, USFR, Schirmer’s test, SGUS, or parotid/glandular swelling, we measured odds ratios and CI and analyzed the data by Fisher’s exact test for the entire study population (n = 105) (**Supplementary Table 1**), JSS patients (n = 27) (**Supplementary Table 2**), and non-JSS patients (n = 78) (**Supplementary Table 3**). No associations were found in all parameters analyzed in those groups. However, there was a negative association found between anti-PSP and parotitis/glandular swelling (**Table 4**) with an odds ratio of 0.068 (CI = 0.005–0.861) in a small group of SSA positive-JSS subjects (n = 19, p = 0.038) compared to an SSA-positive non-JSS group. This negative association was not found between JSS and non-JSS in SSA-negative participants (n = 84, p = 0.174) (**Table 5**).

**Table 4:** Association between diagnostic/glandular items and eSjA in SSA-positive subjects.

Items	eSjA	OR	CI_low	CI_high	p-value
Lip Bx (+: 10 *vs*. :5)	SP1	1	0.068	14.64	1
	CA6	0.667	0.076	5.878	1
	PSP	1.714	0.131	22.513	1
Unstimulated Saliva Flow Rate ( < 0.1:4 *vs*. ≥ 0.1:15)	SP1	1.333	0.1	17.823	1
	CA6	4.5	0.374	54.155	0.303
	PSP	0.917	0.073	11.577	1
Schirmer Test (+:2 *vs*. :7)	SP1	6	0.183	196.28	0.417
	CA6	0.75	0.032	17.506	1
	PSP	0	0	NA	1
SGUS (+:14 *vs*. :5)	SP1	0.25	0.024	2.577	0.272
	CA6	1.5	0.189	11.927	1
	PSP	1.6	0.134	19.09	1
Parotitis or glandular swelling (+:12 *vs*. :7)	SP1	2	0.166	24.069	1
	CA6	0.536	0.081	3.533	0.65
	PSP	0.068	0.005	0.861	0.038*

P-values were derived from Fisher’s exact test on 19 subjects. *p < 0.05; OR, odd ratio; CI, confidence interval.

**Table 5:** Association between diagnostic/glandular items and eSjA in SSA-negative subjects.

Items	eSjA	OR	CI_low	CI_high	p-value
Lip Bx (+:30 *vs*. :28)	SP1	1.042	0.357	3.044	1
	CA6	1.524	0.54	4.297	0.445
	PSP	0.909	0.287	2.877	1
Unstimulated Saliva Flow Rate ( < 0.1:17 *vs*. ≥ 0.1:67)	SP1	0.36	0.094	1.377	0.157
	CA6	1.306	0.444	3.84	0.787
	PSP	1.533	0.513	4.584	0.566
Schirmer Test (+:9 *vs*. :39)	SP1	0.411	0.075	2.239	0.451
	CA6	0.221	0.041	1.201	0.137
	PSP	0.25	0.028	2.218	0.25
SGUS (+:11 *vs*. :54)	SP1	1.053	0.273	4.058	1
	CA6	0.53	0.144	1.959	0.504
	PSP	0.637	0.151	2.683	0.733
Parotitis or glandular swelling (+:39 *vs*. :45)	SP1	1.538	0.618	3.83	0.366
	CA6	2.232	0.926	5.38	0.083
	PSP	1.913	0.764	4.793	0.174

P-values were derived from Fisher’s exact test on 84 subjects. OR, odd ratio; CI, confidence interval.

### A Difference in FS Is Shown Between JSS and Non-JSS in ANA-Positive Individuals While USFR or ESSPRI Dryness/Mean Difference Between the Two Groups Is Found in SP1-Positive or SSA-Positive Participants, Respectively

**Figure 2:**
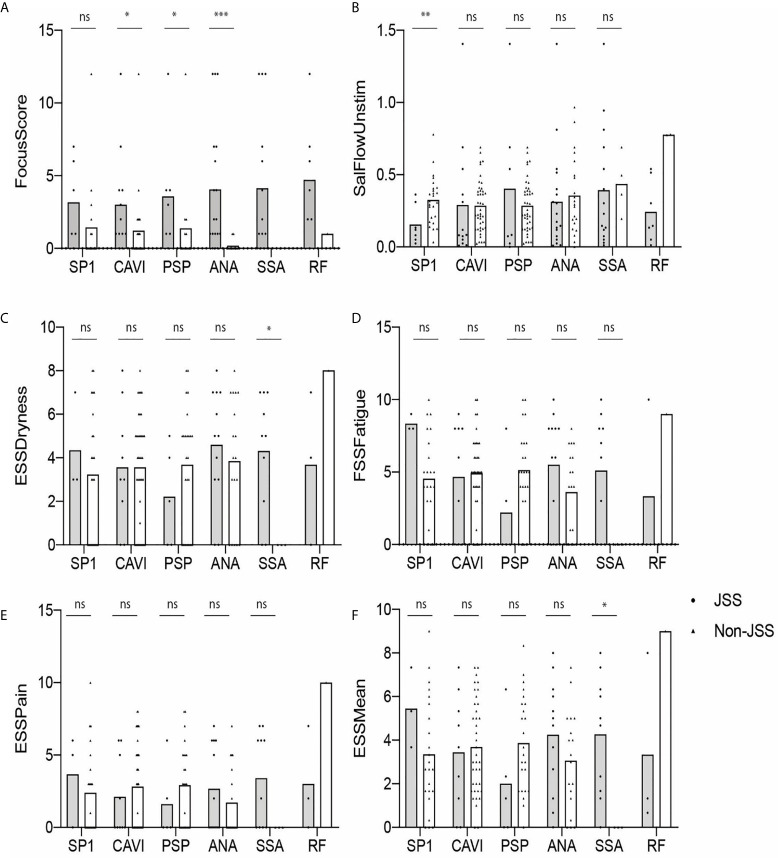
Autoantibody-positive JSS and non-JSS plotted for objective and subjective parameters **(A–F)**. The statistical significance is displayed above the bracket of each bar. The brackets were not depicted and p-values were not calculated when the sample number was below 3 in either group. Each dot depicts actual value of the parameter for each subject. Gray bar, JSS; white bar, non-JSS; ns, not significant; *p ≤ 0.05; **p ≤ 0.01; ***p ≤ 0.001.

We also determined if eSjA or cSjA-positive JSS (gray) and non-JSS (white) showed any differences in the values of glandular associated items, such as FS, USFR, or ESSPRI (**Figure 2** **)**. The JSS group and non-JSS group were significantly different for FS in ANA-positive sub-groups. The difference in FS was also found in anti-CA6-positive JSS or anti-PSP-positive JSS in comparison with anti-CA6 positive or anti-PSP-positive non-JSS patients to a lesser degree, respectively (**Figure 2A**). In addition, USFR was lower in the SP1-positive JSS subgroup compared to the SP1 positive non-JSS group (0.15 vs. 0.32) (**Figure 2B**). SSA-positive subjects reported higher values of ESSPRI Dryness or Mean scores in JSS compared to non-JSS (**Figures 2C–F**). The p-values were calculated only when there was a minimum of three values in each group.

### A Higher FS Is Noted in RF-Positive Participants Than in RF-Negative Subjects

**Figure 3:**
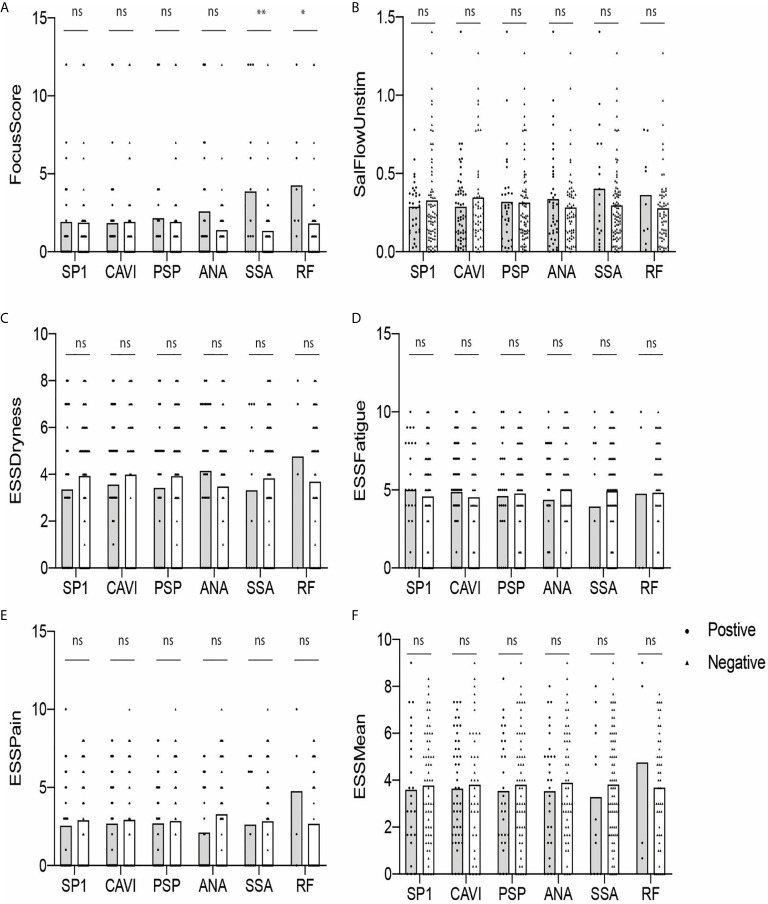
Autoantibody-positive and -negative groups plotted for objective and subjective parameters **(A–F)**. The statistical significance is displayed above the bracket of each bar. The brackets were not depicted and p-values were not calculated when the sample number was below 3 in either group. Each dot depicts actual value of the parameter for each subject. Gray bar, autoantibody positive group; white bar, autoantibody negative group; ns, not significant; *p ≤ 0.05; **p ≤ 0.01.

Next, we determined if there was a difference in the values of FS, USFR, or ESSPRI between antibody-positive (gray) and antibody-negative (white) groups. As shown in **Figure 3**, none of the parameters were statistically different between those two groups except for the RF-positive group compared to the RF-negative group (p < 0.05) (**Figure 3A**). No difference was found in these parameters between eSjA-positive and eSjA-negative individuals.

## Discussion

Considering that immune cell reactivity against glandular antigens is highly likely associated with secretory dysfunction in SS, the discovery of eSjA as a biomarker for early diagnosis is an exciting step toward early intervention. Currently, how autoantibodies against SP1, CA6, and/or PSP antigens are generated or what their exact roles are in SS or JSS remain controversial.

SP1 mRNA was detected in mouse salivary and lacrimal glands (21). The human homologue of SP1 has not been identified, thus necessitating the use of mouse SP1 for the eSjA test kit (13, 22). In a mouse study on the roles of thymus autoimmune regulator gene (AIRE), ectopic expression of SP1 was detected in the thymus (23). Therefore, further investigation is warranted to identify if an emergence of anti-SP1 in SS or other autoimmune conditions signifies failed central tolerance involving AIRE. A high homology of SP1 to a putative lipoprotein of *Clostridium perfringens* raises another possibility that the positivity of anti-SP1 may be related to previous immune exposure to the microflora in respiratory or gastrointestinal tracts (13).

PSP expressed in rodent parotid glands was also detected in human parotid gland and saliva (24). PSP is known to bind and remove infectious agents (25) and targeted knock-out of the gene was sufficient to affect lipopolysaccharide activity, causing mild inflammation (26). Furthermore, a cleaved form of PSP has been implicated in SS‐like autoimmune exocrinopathy in the NOD and C57BL/6.NOD-Aec1Aec2 models (27, 28). CAs catalyze the conversion of CO_2_ to bicarbonate and protons, and CA6 (or CAVI) is the only secreted CA enzyme among 16 α-CA isozymes (29). CA6 is expressed in developing rat sublingual and submandibular glands (30), serous acinar cells of the human parotid and submandibular glands (31), pancreas (32), mammary glands and milk (33), and lacrimal glands (34).

We hypothesized in this study that eSjA against SP1, PSP, and/or CA6 mentioned above might differentiate JSS from non-JSS during early glandular damage that may be present in JSS. Our study found that: 1) JSS patients differed from non-JSS with respect to hypergammaglobulinemia, cytopenias, USFR, MSG lip biopsy, SGUS, history of parotitis/glandular swelling, sicca symptoms (both dry eyes and dry mouth), and dental caries, 2) Potentially applicable levels of sensitivity and specificity of autoantibodies to distinguish JSS from non-JSS were found only in cSjA, 3) Anti-CA6 IgG was the most prevalent eSjA in both groups, but was not specific for JSS. 4) Anti-PSP was negatively associated with parotitis/glandular swelling in the SSA-positive participants, and 5) there was a statistical difference between JSS and non-JSS in FS of ANA-positive subjects, and to a lesser degree, in anti-CA6-positive or anti-PSP-positive subjects. 6) USFR or ESSPRI Dryness/Mean items differed in SP1-positive or SSA-positive JSS groups compared to antibody-positive non-JSS, respectively, 7) FS was higher in the RF-positive subjects than in RF-negative participants.

Since JSS-specific diagnostic criteria are currently unavailable, we applied the 2016 ACR/EULAR SS criteria to JSS diagnosis in this study. As a result, a difference between JSS and non-JSS in MSG lip biopsy results and abnormal serology was anticipated. Interestingly, USFR, SGUS, sicca symptoms, and parotitis/glandular swelling also differed between JSS and non-JSS. Although a common presentation of parotitis in JSS is consistent with other studies (5, 35), our current findings on the high number of JSS patients reporting dry mouth and/or dry eyes disagree with the notion that pediatric patients seldom develop sicca symptoms or altered secretory function (36). Our clinical observations indicate that young children and some of teenagers tend to be unaware or have difficulty in verbalizing sicca symptoms and, therefore, may be underreported. A through history taking and objective measurements of dryness would be vital to recognize children with potential JSS.

Our analysis of ANA revealed a sensitivity of 69.2% and specificity of 70.8%, and the specificity of SSA was high (sensitivity 55.6% and specificity 94.7%). When combinations of autoantibodies are considered, sensitivity tends to increase, but specificity decreases as shown by ANA/SSA (sensitivity 77.8% and specificity 71.8%). Of note, autoantibodies depicted in our best performance cluster consisted of only cSjA without including any eSjA. The performance of eSjA, when any of the nine autoantibodies was positive, showed 55.6% sensitivity and 26.9% specificity, suggesting that eSjA may not be sensitive and specific enough to differentiate JSS from non-JSS as compared to ANA and/or SSA.

Among eSjA, anti-CA6 IgG was most prevalent in both groups. Interestingly, anti-CAII has been implicated in the pathogenesis of autoimmune diseases, such as systemic lupus erythematosus (SLE), SS, autoimmune cholangitis, autoimmune pancreatitis, and recently in Behcet’s disease (37–46). In addition, immunization of mice with human CA II produced sialoadenitis in an MHC-restricted manner (47) and autoantibodies to CA I have also been observed in patients with SS and idiopathic chronic pancreatitis (44). In a study of primary SS with renal manifestations and non-SS sicca patients (48), the levels of anti-CA I, II, VI and VII antibodies were significantly higher in primary SS. Therefore, it would be important to determine if anti-CA6 in eSjA is produced as a consequence of the immune reaction against other CA isoforms that mimic CA6.

There was no associations found between eSjA and diagnostic/glandular items (MSG lip biopsy, SSA, USFR, Schirmer’s test, SGUS, and parotitis/glandular swelling) in our total cohort, JSS, or non-JSS subgroups. Interestingly, in a small number of the SSA-positive group, anti-PSP was negatively associated with parotitis/glandular swelling (p = 0.038 by Fisher’s exact test), whereas no association was found in the SSA-negative group. This finding was interesting because one would predict increased prevalence of glandular tissue-specific autoantibodies such as anti-PSP where there is inflammation in the target tissue (i.e., parotitis or higher FS). However, studies have shown that eSjA tends to be prevalent in patients with negative/low FS, or in SSA-negative individuals (22, 49). Whether these findings signify that eSjA serves as an early antibody for SS/JSS occurring in the absence of overt disease phenotype in the glands or simply indicates non-specificity or lack of a direct link to the degree of glandular damage is still unclear. Therefore, the investigation of eSjA in a larger cohort with long-term follow-up remains highly critical to understanding the clinical significance of eSjA.

Additionally, FS was higher in JSS compared to non-JSS in ANA-positive subjects, and, to a lesser degree, in anti-CA6 or anti-PSP-positive JSS compared to antibody-positive non-JSS. The reduced USFR was found in JSS compared to non-JSS in the SP1-positive subjects. In the SSA positive participants, ESSPRI Dryness and Mean items were all higher in JSS than in non-JSS. However, there were no differences in those parameters between antibody-positive and antibody-negative groups except for the difference in FS found between the RF-positive and -negative groups in our cohort. Interestingly, a recent publication reported worse exocrine function and serologic profile, but not extraglandular manifestations, in adult patients with primary SS with high RF, especially IgA isotype (50). Therefore, investigation of RF in a larger JSS cohort would be worthwhile to determine its potential role in JSS.

Because most studies on eSjA in SS have investigated dry eye, sicca, or primary SS with secondary SS cohorts, a direct comparison among published studies or even with our current study can be challenging to draw a definite conclusion on the clinical validity of eSjA. However, we have recognized some general features of eSjA in SS that have emerged in recent publications.

First, either anti-CA6 or anti-SP1 has been the most prevalent eSjA among the nine items screened for SS. A high prevalence of anti-CA6 was also found in the Sjögren’s International Collaborative Clinical Alliance (SICCA) cohort study (51) as well as in the John’s Hopkins cohort (49). The latter study showed an association of eSjA with severe sicca symptoms, but eSjA did not differentiate SS-dry eye from non-SS dry eye. Similarly, anti-CA6 was most frequently elevated in patients with dry eye condition and a clinical suspicion of SS when a defined population of US veterans in Miami was screened (52). There were no demographic or comorbidity differences between eSjA-positive and eSjA-negative subjects, and eSjA did not correlate with more severe tear film measures. Interestingly, the University of Pennsylvania SICCA cohort study indicated anti-SP1 IgM and anti-PSP IgA as most prevalent, instead of anti-CA6, in SS-dry eye compared to non-SS dry eye (53). The higher prevalence of anti-SP1 was also reported in the SS group (54) and in idiopathic dry eye patients of the (55) of the Dry Eye Assessment and Management (DREAM) cohort. In our study, we found anti-CA6IgG to be the most common without differentiating JSS from non-JSS.

Second, eSjA tends to be positive in participants with negative/low MSG biopsy or with normal salivary secretion as reported in the John’s Hopkins study (49) and the study with a Chinese cohort (22). When Xuan et al. specifically examined anti-SP1 positivity in the latter study, SS patients were strongly positive compared to healthy controls, rheumatoid arthritis (RA), or SLE (22). We did not find any association between eSjA and FS/USFR in this current JSS study. There was no inverse association between eSjA with SSA in our cohort, either, unlike previous studies (13, 55).

Third, clinical associations demonstrated between eSjA and demographic, clinical, or laboratory features are still controversial to date as briefly pointed out earlier. Our study found no association except for anti-PSP, which was negatively associated with history of parotitis/glandular swelling in the SSA-positive JSS group compared to antibody positive non-JSS group while no such association was found in the SSA-negative group. Due to low subject number, significance of this finding warrants further investigation.

Last, eSjA does not appear to differentiate primary SS from secondary SS patients. A cross-sectional study by Shen et al. of the Greek Cohort revealed that anti-SP1 is similarly seen in both primary and secondary SS and rarely in HC. Patients with SS and lymphoma expressed SSA, SSB and anti-SP1 together (22, 56). In a Belgium study (57) conducted by the same group, anti-CA6 IgA was most prevalent (38%) in a cohort with long standing SS. Patients with secondary SS also showed positive eSjA compared to non-SS, but the difference failed to reach statistical significance. Neither SSA nor eSjA was able to distinguish SLE patients with SS from those without. eSjA has also been reported in fibromyalgia patients with sicca and/or xerostomia (22).

Our study was limited by small sample size, which led to ND (not-determined) designation in some of our data analyses. It is also possible that we may have missed some JSS diagnoses since not all 105 participants underwent testing of all five items included in the adult SS-diagnostic criteria, especially ocular criteria, which may not be feasible in some young children. Furthermore, without knowing if JSS is a separate disease entity or if it will ultimately follow the same natural history as SS, how accurately the adult SS diagnostic criteria identify true JSS is unknown. Nevertheless, foundation for the JSS criteria is being developed by the International Childhood SS Workgroup (6) and the Sjögren’s Foundation. Future evaluation of eSjA in the context of the new JSS criteria will be interesting to pursue once the criteria are established.

In conclusion, cSjA, especially ANA and SSA, still serve as relatively sensitive and specific biomarker, for JSS. Long term follow-up of JSS and non-JSS patients will be critical to determine if eSjA could serve as early diagnostic or prognostic markers since our current study is not supportive of their significance in JSS diagnosis. In addition, it is imperative to characterize these autoantibodies for their timing of occurrence, association with other clinical parameters, identification of human SP1 homologue, testing of potential cross-reactivity between CA6 and other CAs, pathological impact on JSS pathogenesis, and specificity for JSS.

## Data Availability Statement

The original contributions presented in the study are included in the article/**Supplementary Material**. Further inquiries can be directed to the corresponding author.

## Ethics Statement

The studies involving human participants were reviewed and approved by the University of Florida Institutional Review Board (IRB). Written informed consent to participate in this study was provided by the participants’ legal guardian/next of kin.

## Author Contributions

Study concept and design: AT, IJ, IB, KB, and SC. Subject recruitment: AT and SC. Data collection: AT, IJ, IB, KB, AA, ST, AS, CB, RM, ME, and SC. Data analysis: IJ, KB, YL, WZ, and SC. Data interpretation: AT, IJ, IB, KB, YL, YK, WZ, and SC. Manuscript draft: AT, IJ, IB, KB, YL, YK, AA, WZ, ST, AS, CB, RM, ME, and SC. Final manuscript: AT, IJ, and SC. All authors contributed to the article and approved the submitted version.

## Funding

This study was supported by the research grants from NIH/NIDCR DE023838 and DE029833, and Sjögren’s Foundation High Impact Research Grant (SC). Funding was provided in part by an unrestricted grant from Research to Prevent Blindness (CB and AS).

## Conflict of Interest

The authors declare that the research was conducted in the absence of any commercial or financial relationships that could be construed as a potential conflict of interest.
