# Supplementary material for: The Diagnostic Performance of Early Sjögren’s Syndrome Autoantibodies in Juvenile Sjögren’s Syndrome: The University of Florida Pediatric Cohort Study
Source: Front Immunol. 2021 Jun 25;12:704193. doi: 10.3389/fimmu.2021.704193 (PMC8267463; doi:10.3389/fimmu.2021.704193)
Supplement: Supplementary file 1 [file DataSheet_1.docx]

**Supplementary Table 1. Association between diagnostic/glandular items and eSjA in the study population**

| **Item** | **eSjA** | **OR** | **CI_low** | **CI_high** | **p-value** |
| --- | --- | --- | --- | --- | --- |
| Lip Bx  (+:40 vs.$-$:33) | SP1 | 0.963 | 0.361 | 2.567 | 1 |
|  | CA6 | 1.326 | 0.526 | 3.343 | 0.64 |
|  | PSP | 1.011 | 0.36 | 2.844 | 1 |
| SSA  (+:19 vs. $-$:84) | SP1 | 0.533 | 0.162 | 1.758 | 0.413 |
|  | CA6 | 0.78 | 0.288 | 2.115 | 0.8 |
|  | PSP | 0.714 | 0.234 | 2.183 | 0.786 |
| Unstimulated Saliva  Flow Rate  ($<0.1:21 vs. \geq0.1:84)$ | SP1 | 0.471 | 0.145 | 1.531 | 0.29 |
|  | CA6 | 1.625 | 0.61 | 4.326 | 0.464 |
|  | PSP | 1.452 | 0.536 | 3.937 | 0.6 |
| Schirmer Test  (+:11 vs. $-$:47) | SP1 | 0.662 | 0.155 | 2.833 | 0.731 |
|  | CA6 | 0.303 | 0.071 | 1.286 | 0.179 |
|  | PSP | 0.213 | 0.025 | 1.823 | 0.259 |
| SGUS  (+:25 vs. $-$:59) | SP1 | 0.571 | 0.198 | 1.652 | 0.444 |
|  | CA6 | 0.633 | 0.247 | 1.622 | 0.35 |
|  | PSP | 0.704 | 0.253 | 1.957 | 0.616 |
| Parotitis or glandular  swelling  (+:52 vs. $-$:53) | SP1 | 1.475 | 0.639 | 3.403 | 0.402 |
|  | CA6 | 1.784 | 0.822 | 3.869 | 0.173 |
|  | PSP | 1.123 | 0.493 | 2.562 | 0.835 |

P-values were derived from Fisher’s exact test on 105 subjects. OR, odd ratio; CI, confidence interval

**Supplementary Table 2. Association between diagnostic/glandular items and eSjA in JSS patients**

| **Item** | **eSjA** | **OR** | **CI_low** | **CI_high** | **p-value** |
| --- | --- | --- | --- | --- | --- |
| Lip Bx  (+:22 vs. $-$:4) | SP1 | 1.125 | 0.097 | 13.036 | 1 |
|  | CA6 | 1 | 0.119 | 8.421 | 1 |
|  | PSP | 1.125 | 0.097 | 13.036 | 1 |
| SSA  (+:15 vs. $-$:12) | SP1 | 0.5 | 0.087 | 2.86 | 0.662 |
|  | CA6 | 0.875 | 0.191 | 3.999 | 1 |
|  | PSP | 1.091 | 0.192 | 6.196 | 1 |
| Unstimulated Saliva  Flow Rate  ($<0.1:11 vs. \geq0.1:16)$ | SP1 | 1.125 | 0.197 | 6.434 | 1 |
|  | CA6 | 2.917 | 0.594 | 14.327 | 0.252 |
|  | PSP | 2.476 | 0.428 | 14.342 | 0.391 |
| Schirmer Test  (+:5 vs. $-$:11) | SP1 | 1.167 | 0.133 | 10.221 | 1 |
|  | CA6 | 0.556 | 0.065 | 4.755 | 1 |
|  | PSP | 0 | 0 | NA | 0.509 |
| SGUS  (+:16 vs. $-$:10) | SP1 | 0.778 | 0.133 | 4.536 | 1 |
|  | CA6 | 0.519 | 0.104 | 2.581 | 0.688 |
|  | PSP | 0.778 | 0.133 | 4.536 | 1 |
| Parotitis or glandular  swelling  (+:19 vs. $-$:8) | SP1 | 3.231 | 0.321 | 32.477 | 0.633 |
|  | CA6 | 0.9 | 0.172 | 4.699 | 1 |
|  | PSP | 0.188 | 0.029 | 1.199 | 0.145 |

P-values were derived from Fisher’s exact test on 27 subjects. OR, odd ratio; CI, confidence interval

**Supplementary Table 3. Association between diagnostic/glandular items and eSjA**

**in non-JSS patients**

| **Item** | **eSjA** | **OR** | **CI_low** | **CI_high** | **p-value** |
| --- | --- | --- | --- | --- | --- |
| Lip Bx  (+:18 vs. $-$:29) | SP1 | 1.209 | 0.358 | 4.089 | 0.766 |
|  | CA6 | 1.538 | 0.471 | 5.023 | 0.556 |
|  | PSP | 1.01 | 0.271 | 3.757 | 1 |
| SSA  (+:4 vs. $-$:72) | SP1 | 0.667 | 0.066 | 6.754 | 1 |
|  | CA6 | 0.846 | 0.113 | 6.341 | 1 |
|  | PSP | 0.627 | 0.062 | 6.342 | 1 |
| Unstimulated Saliva  Flow Rate  ($<0.1:10 vs. \geq0.1:68)$ | SP1 | 0.204 | 0.024 | 1.706 | 0.155 |
|  | CA6 | 1.333 | 0.345 | 5.152 | 0.745 |
|  | PSP | 1.394 | 0.357 | 5.449 | 0.724 |
| Schirmer Test  (+:6 vs. $-$:36) | SP1 | 0.354 | 0.037 | 3.364 | 0.645 |
|  | CA6 | 0.16 | 0.017 | 1.511 | 0.184 |
|  | PSP | 0.4 | 0.042 | 3.818 | 0.647 |
| SGUS  (+:9 vs. $-$:49) | SP1 | 0.492 | 0.092 | 2.628 | 0.476 |
|  | CA6 | 0.862 | 0.206 | 3.613 | 1 |
|  | PSP | 0.861 | 0.192 | 3.87 | 1 |
| Parotitis or glandular  swelling  (+:33 vs. $-$:45) | SP1 | 1.407 | 0.539 | 3.668 | 0.624 |
|  | CA6 | 2.5 | 0.984 | 6.352 | 0.067 |
|  | PSP | 2.026 | 0.779 | 5.269 | 0.155 |

P-values were derived from Fisher’s exact test on 78 subjects. OR, odd ratio; CI, confidence interval OR, odd ratio; CI, confidence interval
